# Metabolomics Plasma Biomarkers Associated with the HRD Phenotype in Ovarian Cancer

**DOI:** 10.3390/metabo16010002

**Published:** 2025-12-19

**Authors:** Alessandro Tubita, Claudia De Angelis, Daniela Grasso, Flavia Sorbi, Francesca Castiglione, Lorenzo Anela, Maria Cristina Petrella, Massimiliano Fambrini, Federico Scolari, Andrea Bernini, Giulia Petroni, Serena Pillozzi, Lorenzo Antonuzzo

**Affiliations:** 1 Department of Experimental and Clinical Medicine, University of Florence, 50134 Florence, Italy; alessandro.tubita@unifi.it (A.T.); lorenzo.anela@unifi.it (L.A.); massimiliano.fambrini@unifi.it (M.F.); giuliapetroni@gmail.com (G.P.); lorenzo.antonuzzo@unifi.it (L.A.); 2 Oncology Unit, Careggi University Hospital, 50134 Florence, Italy; deangeliscl@aou-careggi.toscana.it (C.D.A.); petrellamc@aou-careggi.toscana.it (M.C.P.); 3 Department of Biotechnology, Chemistry and Pharmacy, University of Siena, 53100 Siena, Italy; daniela.grasso@student.unisi.it (D.G.); andrea.bernini@unisi.it (A.B.); 4 Department of Experimental and Clinical Biomedical Sciences, University of Florence, 50134 Florence, Italy; flavia.sorbi@unifi.it (F.S.); federico.scolari@unifi.it (F.S.); 5 Department of Health Sciences, University of Florence, 50134 Florence, Italy; castiglionefr@aou-careggi.toscana.it

**Keywords:** ovarian cancer, biomarker, liquid biopsy, homologous recombination deficiency (HRD), BRCA1/2 mutations

## Abstract

**Background**: Ovarian cancer (OC) remains one of the most lethal gynecologic malignancies due to its often-late diagnosis and complex molecular heterogeneity. Understanding the metabolic alterations in OC can provide insights into its pathophysiology and potential therapeutic targets. This study aimed to explore serum metabolomic profiles and their correlation with clinical and pathological features in OC patients. **Materials and Methods**: Thirty serum samples were collected from patients diagnosed with ovarian tumors (OTs) (n = 24 malignant, n = 6 benign) and undergoing treatment at Careggi University Hospital. Additionally, 47 samples were obtained from age-matched healthy female donors. Serum samples underwent processing and analysis using an H-NMR (Nuclear Magnetic Resonance) platform to identify a panel of metabolites. Correlation analysis between the metabolomic data and clinical parameters was performed using R software (v.4.4.0). **Results**: Differential metabolomic profiling showed a significant upregulation of metabolites associated with the purine salvage pathway (i.e., hypoxanthine and inosine) and the ketone bodies axis (i.e., acetone, 3-hydroxybutyrate, and acetate) in samples from ovarian tumor (OT) patients compared to healthy donors. Within malignant OC samples, metabolomic profiles significantly correlated with BRCA1/2 mutation status (BRCA1/2-mutated vs. wild-type) and homologous recombination deficiency (HRD) status. **Conclusions**: The analysis revealed significant variation in specific metabolites such as betaine, creatinine, carnitine, glycerol, and mannose; notably, a downregulation of these metabolites was observed in HRD-positive patients. The study identifies significant metabolomic alterations in OC, implicating pathways such as purine salvage and ketone bodies. Intriguingly, consistent variation in specific metabolites across BRCA/HRD phenotypes underscores their potential as OC biomarkers. Further research is needed to validate these findings and explore their prognostic and therapeutic implications.

## 1. Introduction

Ovarian cancer (OC) represents the leading cause of death among gynecological cancers and the fifth most frequent cause of death in women, in general [1,2]. Despite extensive research efforts, the prognosis of ovarian cancer (OC) remains poor, primarily due to late-stage diagnosis [3]. Approximately 80% of OC cases are identified at advanced stages (III/IV), with a 5-year survival rate of 10 to 40%. In contrast, when diagnosed at an early stage, about 20% of patients have a markedly improved 5-year survival rate of 70% to 95% [3]. Epithelial ovarian carcinomas (EOCs) represent approximately 90% of ovarian cancer cases, with high-grade serous carcinoma (HGSOC; 70%) as the most prevalent subtype, followed by high-grade endometrioid (HGEOC; 10%), clear cell (6–10%), low-grade serous (~5%), and mucinous (3–4%) carcinomas [4]. About 50% of OCs exhibit mutations in the genes involved in the homologous recombination repair. A total of 20% of mutations occur in BRCA1/2 genes with 14% being germline (gBRCA) and 6% somatic (sBRCA). The remaining alterations (mutations, deletions, amplifications) involve genes such as CDK12, RAD51C, PALB2, FANC, CHEK2, PTEN, and EMSY, classifying these tumors as homologous recombination deficiency (HRD)-positive [5]. Actually, debulking surgery, either “upfront” or “interval”, plays a key role in the management of OC patients irrespective of disease stage. Platinum-based chemotherapy, essentially represented by the combination of carboplatinum and paclitaxel, is employed either as adjuvant in early stages and as neoadjuvant/first-line therapy or at relapse in advanced stages [4,6,7]. In advanced stages, maintenance therapy following platinum-based chemotherapy is primarily based on the use of poly(ADP-ribose) polymerase (PARP) inhibitors—olaparib (with or without bevacizumab), rucaparib, and niraparib—guided by BRCA1/2 mutation and HRD status [8,9,10,11,12,13,14]. HRD-positive status is the most prevalent alteration in OC, reaching up to 69% by The Cancer Genome Atlas (TCGA) when all types of alterations are considered, germline and somatic mutations, genomic scoring, epigenetic changes, and gnomic instability [15]. HRD is a common feature of HGSOC, conferring a favorable prognosis with better long-term outcomes and increased sensitivity to platinum-based therapies and PARP inhibitors [9,16].

The analysis of HRD has taken on a central role in guiding optimal first-line treatment strategies. Because cancer cells are able to support their growth, proliferation, invasion, and metastasis through reprogramming their energy metabolism, the discovery of new cancer biomarkers in the effort of personalized treatment is crucial. In OC, global metabolomic studies have emerged over the past decades [17,18]. However, the current literature clearly demonstrates that the application of metabolomics in the clinical management of OC is still at a very early stage [17,18].

The aim of our study is to evaluate the metabolomic profile of a cohort of women diagnosed with malignant OC in general and, more specifically, of those patients exhibiting homologous recombination deficiency (HRD) or BRCA1/2 mutations [19].

## 2. Materials and Methods

### 2.1. Patients

Thirty serum samples (24 malignant and 6 benign) collected between 2019 and 2022 from patients with OT and followed up at Careggi University Hospital were obtained (Table 1). A total of 47 serum samples were obtained from healthy female controls, age-matched to the patients. Clinical, pathological, and genetic data were obtained by retrospective chart abstraction. The treatment administered was given per institutional guidelines. Ethical approval was granted by the institutional ethics committee (CEAVC n. 14780). Informed consent for the research use of the samples and clinicopathological data was obtained according to these approvals by all patients.

### 2.2. Sample Materials and Sample Preparation

Dipotassium hydrogen phosphate (K_2_HPO_4_), monopotassium phosphate (KH_2_PO_4_), calcium formate, sodium hydroxide, and D_2_O have been purchased from Merck KGaA (Darmstadt, Germany). Deionized water was purified using a Milli-Q^®^ System from Millipore (Billerica, MA, USA). From 2019 to 2022, 30 ovarian tumor (OT) serum samples (24 malignant and 6 benign) were obtained from patients affected by OT and followed up at Careggi University Hospital. A total of 47 serum samples were obtained from healthy female controls to match the patients’ age. Clinical and pathological data were obtained by retrospective chart abstraction. The treatment administered was given per institutional guidelines at the time of diagnosis. Ethical approval was received from the institutional ethics committee. Informed consent for the research use of the samples and clinicopathological data was obtained according to these approvals by all patients. A stock solution of phosphate buffer 500 mM (PB) was prepared from KH_2_PO_4_ and K_2_HPO_4_, and pH was adjusted to 7.0 with NaOH 1 M. Serum samples were prepared with 300 μL of serum, 60 μL formate, 60 μL PB, and 80 μL H_2_O to final concentrations of 4.5 mM formate and 50 mM PB. Formate stock solution 45.0 mM was prepared in deuterated water in order to obtain a final 10% D_2_O concentration for locking and shimming.

### 2.3. ^1^H-NMR Spectroscopy

All experiments were performed on a Bruker Avance™ 600 spectrometer (Bruker Biospin AG, Fällanden, Switzerland) operating at 14.1 T. A PROJECT (Periodic Refocusing of J Evolution by Coherence Transfer) pulse sequence (an optimized-CPMG pulse sequence; [20]) was used with an echo time (τ) of 0.3 ms with 128 loops to obtain a T2 filter delay of 153.6 ms [21,22]. To allow full magnetization recovery for the compound, an additional delay (20 s) was added to T2 pulse sequences before repetition/presaturation delay [23]. The NMR data were processed with TopSpin 4.0.8 software and analyzed with Chenomx 10 (Chenomx Inc., Edmonton, CA, USA). All the 1D proton spectra were acquired with a spectral width of 10 kHz. All spectra were obtained with 16 scans, digitalized over 32 k points, and zero-filled to 128 k points. Solvent signal removal was achieved with presaturation power of 55 dB during delay d1 (4 s).

### 2.4. Statistical Analysis

Clinical data are reported as absolute numbers and percentages. The *p* values were calculated using student *T*-test (two groups). A *p* < 0.05 was considered statistically significant. Statistical analysis has been performed using MetaboAnalyst (v.6.0) [24]. Multidimensionality reduction has been achieved using partial least squares discriminant analysis (PLS-DA), reporting the first two principal components. Significant metabolite variations have been assessed using a combination of fold change (−0.5 > log_2_FC > 0.5) and *p* value (−log_10_P > 1.30) thresholds.

## 3. Results

A total of 30 patients affected by OTs have been enrolled. Of these, 6 patients were diagnosed with benign OCs (OCB), while 24 patients were diagnosed with malignant OCs (OCM) with a median age of 65 years (range 44–83) (Table 1).

In patients with OCB, the mean age was 46 years. The most representative histological types were mucinous cystadenoma and mucinous borderline tumors, each accounting for 33% of the cases. The median BMI in all OCB patients was below 25, and blood glucose levels were consistently below 100 mg/dL. Moreover, patients were classified according to menopausal status: 67% were premenopausal and 33% postmenopausal.

Among the OCMs, 17 (71%) patients were diagnosed at advanced stages (FIGO III–IV), while 7 (29%) were diagnosed at early stages (FIGO I–II). The histological subtypes identified were as follows: 71% HGSOC, 13% HGEOC, 8% mucinous OC, 8% other histotypes. Among the twenty patients with BRCA1/2 mutations status known, three (15%) patients exhibited germline gBRCA1/2 mutations and three (15%) exhibited somatic sBRCA1/2 mutations. Two cases (10%) were identified as HRD-positive and had a family history of breast or ovarian cancer. However, the specific gene mutations could not be determined, as HRD status was assessed using the Myriad myChoice^®^ test, which does not provide information on the individual genes involved. Two patients reported a personal history of breast cancer, with only one carrying a germline BRCA1/2 mutation. All 17 (71%) OC patients at stage III–IV received first-line platinum-based chemotherapy. Four (17%) patients having germline or somatic BRCA1/2 mutation received Olaparib as maintenance to first-line chemotherapy; two (8%) HRD-positive patients received Olaparib plus bevacizumab as maintenance; one (4%) patient with sBRCA1/2 mutations received bevacizumab as maintenance; and eight (33%) patients without BRCA1/2 mutations or HRD-positivity received alternative maintenance to first-line chemotherapy: five (21%) patients received Niraparib and three (13%) patients received bevacizumab.

Median BMI was 24.3 (range 18.3–33.7). Ten patients had a BMI ≥ 25 with one classified as obese. Four (17%) patients showed a blood glucose level > 100 mg/dL. Four (17%) patients were premenopausal at diagnosis. Three (13%) patients had diabetes type 2, five (21%) patients had hypercholesterolemia/hypertriglyceridemia, five (21%) patients had hypertension, four (17%) patients had hypothyroidism, and three (13%) patients had a thromboembolism disorder. Medication use included antihypertensive drugs in ten patients (42%), statins or ezetimibe in five patients (21%), antidepressants in five patients (21%), and proton pump inhibitors in three patients (13%).

### 3.1. NMR Analysis on Basal Serum Samples of OT Patients and Healthy Donors

**Figure 1:**
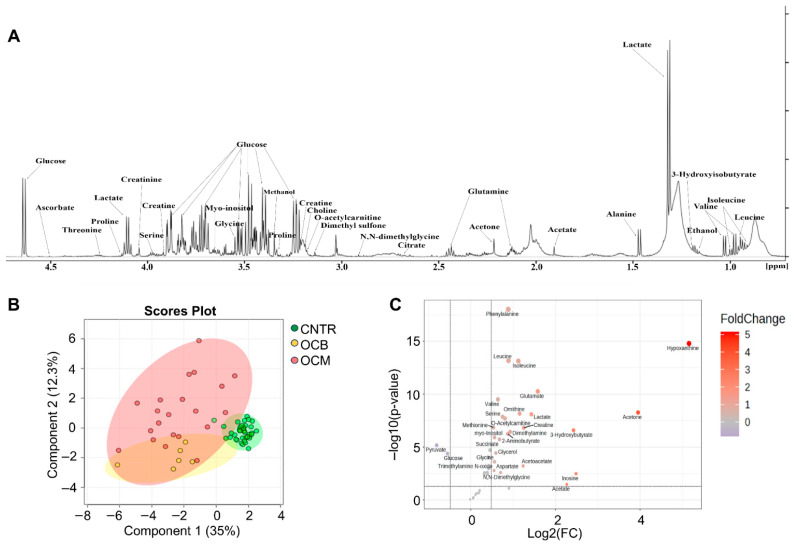
(**A**) Hydrogen 1D spectrum (aliphatic region) of serum sample from an OC patient. Signals of some relevant metabolites among those identified are labeled. All the identified metabolites were quantified using the Chenomx NMR software (v 8.2; Edmonton, AB, Canada). (**B**) PCA scores plot obtained from 30 OT serum samples (24 malignant and 6 benign) and 47 serum samples from healthy female controls. Benign ovarian cancer (yellow; OCB) and malignant ovarian cancer samples (red; OCM) vs. healthy controls samples (green; CNTR) are indicated by dots. The score plot shows that samples from serum samples are clustered separately, with the model explaining 59.7% of the variance in X. (**C**) Volcano plot highlights the metabolites that have a fold change (FC) of over 1.4 (log_2_(FC) = 0.3) and a *p* < 0.05.

**Figure 2:**
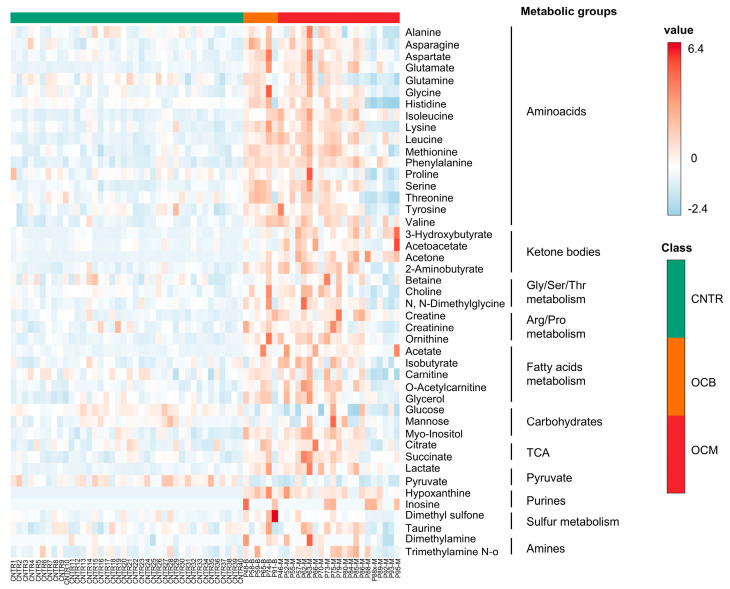
Heatmap showing changes in the levels of 45 metabolites, grouped by metabolic pathways, in benign ovarian cancer (OCB) and malignant ovarian cancer (OCM) compared to the control group (CNTR). Red indicates upregulation; blue indicates downregulation.

NMR investigation was performed on serum samples collected at the diagnosis of OT and on serum samples from healthy donors matched for age. Spectral peak assignment allowed for 45 metabolites to be assigned and quantified (see Figure 1A as an example of aliphatic groups assignments). The PCA score plot shows a clear separation between the two groups of samples examined, suggesting a characteristic metabolomic profile for each group. In fact, the principal component (PC) 1, accounting for over 35% of the total variance of the dataset, allows for a net separation of OT patients from controls (CNTR); PC2 distribution is notably broader for OCMs with a positive axis distribution, while for OCB it also exhibits distinct separation; the PC3 only explains 4.5% of the existing variance (Figure 1B). The Volcano plot (Figure 1C) highlights the metabolites that have a fold change (FC) of over 1.4 (log_2_FC 0.3) and a *p* < 0.05. The five most upregulated metabolites identified in OT samples, compared to healthy donor samples, include hypoxanthine (averages, uM: CNTR: 0.2 vs. OC: 17.7, *p* < 0.00001) and inosine (averages, uM: CNTR: 0.1 vs. OC: 2.5, *p* < 0.001), which are involved in purine metabolism, and acetone (averages, uM: CNTR: 10.8 vs. OC: 167.2, *p* < 0.001), 3-hydroxybutyrate (averages, uM: CNTR: 44.9 vs. OC: 253.4, *p* < 0.00001), and acetoacetate (averages, uM: CNTR: 16.6 vs. OC: 32.3, *p* < 0.001), which are related to ketone bodies metabolism. Importantly, the heatmap shows a net difference between the CNTR and OCB/OCM groups. Notably, the OCM group appears to be divided into two subgroups, where a small subset of patients (the rightmost group of patients P95, P93, P92, P89, P88, and P88*) shows amino acid depletion (Figure 2). In conclusion, both benign and malignant ovarian tumors exhibit a distinct metabolite expression or profile compared to healthy controls, particularly involving purines and ketone bodies.

### 3.2. Metabolic Profile in Malignant OC Patients

By selectively analyzing the metabolomic profile of the malignant OC patient subgroup, we correlated the statistically significant metabolites with clinicopathological parameters (Table 2).

**Figure 3:**
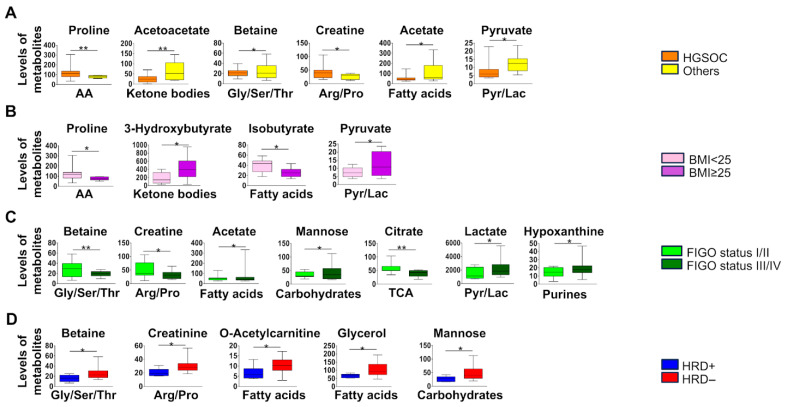
Metabolite level distribution in malignant ovarian cancer (OC) patients. (**A**–**D**) Box plot representations of significant metabolite levels for different clinicopathological parameters (histology, BMI, FIGO status, and HRD status). Data are presented as relative abundances, and statistical significance is calculated by student *T*-test and indicated by asterisks (* *p* < 0.05; ** *p* < 0.01).

Regarding amino acids, elevated levels of proline were associated with high-grade serous carcinoma (HGSOC) and were present in patients with a body mass index (BMI) less than 25 (Figure 3A,B and Table 2). The most significant differences among ketone bodies concern acetoacetate, which showed higher levels in the non-HGSOC group (Figure 3A), and 3-hydroxybutyrate, which was more abundant in patients with a BMI more than 25 (Figure 3B). Subsequently, we provided a detailed analysis of the glycine, serine, and threonine (Gly/Ser/Thr) metabolic group in the context of clinical data. A significant increase in betaine levels in non-HGSOC subtypes and in patients at stage I/II has been noted, while its levels did not appear to be influenced by BMI (Figure 3A–D; Table 2). Early-stage OC has heightened creatine metabolism which is likely to meet the energy needs of rapidly proliferating tumor cells. We also observed an increase in creatine metabolism in the HGSOC subtype (Figure 3A). Concerning ketone body concentrations, significant increases in 3-hydroxybutyrate and acetoacetate levels in non-HGSOC subtypes and in patients with a BMI greater than 25, respectively, have been observed (Figure 3A,B). Another important group of metabolites in OC was represented by fatty acids. Increased levels of acetate in non-HGSOC subtypes were found, and this increase also occurs in advanced-stage OC patients. The next two metabolic groups analyzed were those of carbohydrate metabolism and the tricarboxylic acid (TCA) cycle (also known as the citric acid cycle or Krebs cycle), being pivotal for cancer cell survival and proliferation. In our analyses, we found that the mannose was upregulated in advanced-stage OC patients (Figure 3C), while glucose and myoinositol were not significantly impacted. At the same time, the citrate, belonging to the TCA metabolic group, was upregulated in early-stage OC patients with respect to advanced-stage patients (Figure 3C). Lactate levels were significantly increased in patients at stage III/IV (Figure 3C), while pyruvate was upregulated in non-HGSOC subtypes and in patients with a BMI greater than 25 (Figure 3A,B). Importantly, hypoxanthine appears, within the malignant group, to be characteristic of advanced-stage OC (Figure 3C).

Finally, no significant changes were observed in the last two groups of metabolites analyzed, amines and sulfur metabolic groups, in relation to the clinical parameters (Table 2).

### 3.3. Metabolic Profile in HRD-Positive Malignant OC Patients

Significant low levels of betaine in BRCA1/2-mutated/HRD-positive OC patients were detected. BRCA1/2 wild-type /HRD-negative tumors showed higher creatinine levels compared to corresponding BRCA1/2-mutated/HRD-positive. Patients with HRD- negative status also showed an increase in carnitine and glycerol levels (Figure 3D). In our analyses, we found that the mannose was upregulated in OC patients being BRCA1/2 wild-type/HRD-negative (Figure 3C,D), while glucose and myoinositol were not significantly impacted.

## 4. Discussion

This study provides novel insights into the serum metabolic landscape of patients diagnosed with malignant OC, with a particular focus on the subgroup characterized by HRD status. Using NMR-based metabolomics, we identified distinct metabolic alterations that distinguish OC patients from healthy controls and differentiate subtypes based on histological, molecular, and clinical parameters. The PCA and heatmap analysis revealed a clear metabolic separation between the patients and healthy individuals, with a subset of malignant patients showing amino acid depletion, possibly indicating a more aggressive phenotype. Key altered metabolites included purine derivatives (hypoxanthine, inosine) and ketone bodies (acetone, 3-hydroxybutyrate, acetate), reflecting enhanced nucleotide turnover and altered energy metabolism—hallmarks of malignancy [25].

When analyzing metabolic changes in relation to histological subtypes in malignant cancer cells, we observed elevated proline levels in patients with HGSOC, particularly in those with a BMI < 25. Proline accumulation has been previously linked to collagen remodeling and tumor progression in epithelial cancers, and our findings suggest that it may also serve as a biomarker for HGSOC [26]. In contrast, non-HGSOC subtypes showed increased levels of acetoacetate and acetate, highlighting the potential metabolic divergence based on tumor histotype. Furthermore, ketone body levels, notably 3-hydroxybutyrate, were higher in patients with BMI ≥ 25, aligning with prior studies that associate adiposity with altered ketogenesis and cancer progression [27]. Interestingly, early-stage OC (FIGO I/II) demonstrated enhanced creatine metabolism. Creatine was also elevated in HGSOC patients, reinforcing the aggressive metabolic profile of this subtype. The glycine/serine/threonine pathway also yielded important differences: betaine was upregulated in early-stage and non-HGSOC cancers but significantly depleted in HRD-positive/BRCA1/2-mutated tumors.

HRD and BRCA status were associated with specific metabolic fingerprints. Patients with BRCA1/2 mutations or HRD-positive status had reduced levels of betaine, creatinine, mannose, glycerol, and o-acetyl-carnitine, while wild-type/HRD-negative cases showed elevated levels of these metabolites. Betaine, a key component of one-carbon metabolism, influences DNA synthesis, repair, and methylation through the regulation of homocysteine levels. Elevated homocysteine levels are implicated in DNA damage and oxidative stress, factors associated with cancer risk. While betaine supplementation can lower homocysteine levels and a meta-analysis suggests an inverse correlation between betaine intake and cancer risk, other studies, specifically in OC, have found no significant associations [28,29,30]. In our study, we observed higher betaine levels in HRD-negative OC. Importantly, lower betaine in HRD-positive tumors may impair homocysteine remethylation, leading to aberrant DNA methylation, increased DNA damage, and genomic instability consistent with the HRD-positive phenotype.

The relationship between creatinine and cancer is multifaceted, influenced by factors such as muscle mass and renal function. Serum creatinine levels have been identified as potential prognostic markers in OC, with both high and low levels linked to poorer outcomes, potentially reflecting both renal function and broader metabolic processes [31,32]. Furthermore, the impact of creatine supplementation on cancer is debated, with some studies suggesting tumor growth suppression and enhanced anti-tumor immunity [33], while others suggest the promotion of invasion and metastasis [34]. CKB, a form of creatine kinase, is upregulated in some cancers, including OC, and its suppression has been linked to reduced cancer cell survival [35]. O-acetyl-carnitine, which is involved in the energy and anti-inflammatory pathways, may have anti-tumor effects, although its role in HRD-positive OC requires further investigation. Studies have shown that the downregulation of carnitine acetyltransferase (CRAT) is associated with poorer survival in OC, while the forced expression of CRAT suppresses OC growth and metastasis by inducing cell cycle arrest and inhibiting EMT, potentially through the effects on mitochondrial functions and EMT [36]. The scientific literature highlights a connection between glycerol metabolism and ovarian cancer, although it is not yet fully understood. Derived from triglyceride breakdown, glycerol can fuel gluconeogenesis or lipid synthesis, supporting tumor growth. Glycerol-3-phosphate (G3P), a key intermediate [37], acts as a precursor for essential components of cell membranes and signaling pathways, promoting metastasis, particularly in fat-rich environments. Alterations in enzymes regulating G3P metabolism, such as Glycerol-3-phosphate acyltransferase (GPAM) and Glycerophosphodiesterase EDI3 (GPCPD1/EDI3), have been associated with changes in cellular behavior and prognosis. Notably, higher GPAM expression, which correlates with lower intracellular G3P, has been significantly linked to shorter overall survival in ovarian cancer across multiple datasets [38]. Finally, recent metabolomic studies have shown that serum mannose levels are elevated in patients with OC, especially in advanced stages. Notably, a 2023 study found that the glucose-to-mannose (G/M) ratio could discriminate, as a non-invasive metabolic biomarker, advanced OC patients from healthy controls with high sensitivity and specificity (AUC: 0.98, sensitivity: 91.46%, specificity: 98.50%; [39]). Additionally, increased mannose in advanced-stage and HRD-negative tumors could reflect enhanced glycosylation activity, which has been implicated in tumor immune evasion and metastasis [40,41]. Collectively, our findings demonstrate that serum metabolomics can reveal biologically relevant distinctions among OC subtypes and may contribute to the improved stratification of patients for personalized therapies. Importantly, HRD and BRCA1/2 mutation status appear to significantly influence systemic metabolism, offering a potential avenue for identifying metabolic vulnerabilities in these genetically defined subgroups.

## 5. Conclusions

This study demonstrates that NMR-based serum metabolomics offers valuable insights into the biological heterogeneity of ovarian cancer and may serve as a promising tool for patient stratification. The metabolic profile clearly distinguishes ovarian tumors—both benign and malignant—from healthy controls and reveals specific alterations associated with histological subtype, disease stage, BMI, and, importantly, BRCA1/2 and HRD status. Changes in metabolites related to purine turnover, ketone body metabolism, amino acids, and carbohydrate and lipid pathways indicate extensive metabolic reprogramming that reflects the energetic and biosynthetic demands of tumor cells. Notably, HRD-positive/BRCA-mutated tumors exhibit a distinct metabolic signature characterized by reduced levels of betaine, creatinine, glycerol, and o-acetyl-carnitine, highlighting the potential biological vulnerabilities and candidate biomarkers for this subgroup. Overall, our findings support the potential of metabolomics as a complementary approach to precision oncology, enhancing the understanding of tumor progression and molecular diversity in ovarian cancer. However, the study is limited by its small sample size—especially within specific molecular subgroups—as well as its single-center, retrospective design, which may restrict its generalizability. The absence of an external validation cohort and the lack of functional experiments further limit causal interpretation. Additionally, the heterogeneity of ovarian cancer histotypes and the constraints of HRD testing reduce the precision of subtype-specific conclusions. Further studies with larger cohorts and functional validation are warranted to confirm the clinical utility of these metabolic markers, especially in the context of treatment response and resistance to maintenance therapies such as PARP inhibitors.

## Figures and Tables

**Table 1:** Clinicopathological characteristics of patients with malignant OC. Abbreviations: HGSOC: high-grade serous ovarian cancer, HGEOC: high-grade endometrioid, OC: ovarian cancer, gBRCA1/2: germline BRCA1/2, sBRCA1/2: somatic BRCA1/2, HRD: homologous recombination deficiency, BMI: body mass index, PPI: proton pump inhibitors. * Analysis performed on 20 patients with BRCA1/2 mutations status known.

	Total Number Pts (%) = 24 (100%)
**Age (years), median (range)**	65 (84–43)
**Stage**	
**I–II**	7 (29%)
**III–IV**	17 (71%)
**Histology**	
**HGSOC**	17 (71%)
**HGEOC**	3 (13%)
**Mucinous OC**	2 (8%)
**Other histotypes**	2 (8%)
BRCA1/2 mutations/HRD-positive *	
**Yes**	**8** (40%)
**gBRCA1/2 mutations**	3 (15%)
**sBRCA1/2 mutations**	3 (15%)
**HRD-positive**	2 (10%)
**No**	**12** (60%)
**First-line Chemotherapy**	17 (71%)
**Yes**	**15** (63%)
**Olaparib**	4 (17%)
**Olaparib plus bevacizumab**	2 (8%)
**Niraparib**	5 (21%)
**Bevacizumab**	4 (17%)
**No**	**2** (8%)
**BMI, median (range)**	24.3 (18.3–33.7)
**BMI ≥ 25**	10 (42%)
**BMI < 25**	14 (58%)
**Blood glucose level > 100 mg/dL**	4 (17%)
**Blood glucose level ≤ 100 mg/dL**	20 (83%)
**Premenopausal**	4 (17%)
**Postmenopausal**	20 (83%)
**Comorbidities**	
**Diabetes type 2**	3 (13%)
**Hypercholesterolemia/Hypertriglyceridemia**	5 (21%)
**Hypertension**	5 (21%)
**Hypothyroidism**	4 (17%)
**Thromboembolism disorder**	3 (13%)
**Drugs**	
**Metformin/SGL2 inhibitor**	3 (13%)
**Antihypertensive**	10 (42%)
**Statins/ezetimibe**	5 (21%)
**β-blockers**	2 (8%)
**Levothyroxine**	2 (8%)
**PPI**	3 (13%)
**Antidepressants**	5 (21%)
**Antihormonal therapy**	1 (4%)
**Bisphosphonates**	1 (4%)
**IFN-γ**	1 (4%)
**Immunosuppressants**	1 (4%)
**Antiaggregants**	2 (8%)
**Anticoagulants**	2 (8%)

**Table 2:** Serum metabolites associated with clinicopathological features in ovarian cancer patients.

	FIGO-STATUS	HISTOLOGY	BRCA/HRD	BMI
**AA**		Proline		Proline
**Ketone bodies**		Acetoacetate		
**Ketone bodies**				3-Hydroxibutirate
**Gly/Ser/Thr**	Betaine	Betaine	Betaine	
**Arg/Pro**	Creatine	Creatine		
**Arg/Pro**			Creatinine	
**Fatty acids**	Acetate	Acetate		
**Fatty acids**			O-Acetyl-Carnitine	
**Fatty acids**			Glycerol	Isobutirate
**Carbohydrates**	Mannose		Mannose	
**TCA**	Citrate			
**Pyr/Lac**	Lactate			
**Pyr/Lac**		Pyruvate		Pyruvate
**Purines**	Hypoxanthine			

## Data Availability

Data are available from the authors upon reasonable request.

## Abbreviations

The following abbreviations are used in this manuscript:

BMI	body mass index
CNTR	control
CT	chemotherapy
FC	fold change
HGEOC	high-grade endometroid carcinoma
HGSOC	high-grade serous carcinoma
H-NMR	nuclear magnetic resonance
PCD	programmed cell death
HRD	homologous recombination deficiency
OC	ovarian cancer
OCB	benign ovarian cancer
OCM	malignant ovarian cancer
OT	ovarian tumor
PC	principal component
PLS-DA	partial least squares discriminant analysis
